# Loss of BAP1 Expression Is Very Rare in Pancreatic Ductal Adenocarcinoma

**DOI:** 10.1371/journal.pone.0150338

**Published:** 2016-03-16

**Authors:** Michael Tayao, Juliana Andrici, Mahtab Farzin, Adele Clarkson, Loretta Sioson, Nicole Watson, Terence C Chua, Tamara Sztynda, Jaswinder S Samra, Anthony J Gill

**Affiliations:** 1 Cancer Diagnosis and Pathology Research Group, Kolling Institute of Medical Research, St Leonards, NSW, Australia, 2065; 2 School of Life Sciences, University of Technology Sydney, Ultimo, NSW, Australia, 2007; 3 Sydney Medical School, University of Sydney, Sydney, NSW, Australia, 2006; 4 Department of Anatomical Pathology, Royal North Shore Hospital, St Leonards, NSW, Australia, 2065; 5 Department of Gastrointestinal Surgery, Royal North Shore Hospital, St Leonards, NSW, Australia, and Discipline of Surgery, University of Sydney, Sydney, NSW, Australia; 6 Macquarie University Hospital, Macquarie University, North Ryde, NSW, Australia; 7 Sydney Vital Translational Research Centre, Royal North Shore Hospital, Pacific Highway, St Leonards, NSW, Australia, 2065; University of Nebraska Medical Center, UNITED STATES

## Abstract

**Background:**

Pancreatic cancer is both common and highly lethal and therefore new biomarkers or potential targets for treatment are needed. Loss of BRCA associated protein-1 (BAP1) expression has been found in up to a quarter of intrahepatic cholangiocarcinomas. Given the close anatomical relationship between intrahepatic cholangiocarcinoma and pancreatic ductal adenocarcinoma, we therefore sought to investigate the frequency of loss of BAP1 expression in pancreatic ductal adenocarcinoma.

**Methods:**

The records of the department of Anatomical Pathology Royal North Shore Hospital, Sydney, Australia, were searched for cases of pancreatic ductal adenocarcinoma diagnosed between 1992 and 2014 with material available in archived formalin fixed paraffin embedded tissue blocks. Immunohistochemistry for BAP1 was performed on tissue microarray sections and if staining was equivocal or negative it was confirmed on whole sections. Negative staining for BAP1 was defined as loss of expression in all neoplastic nuclei, with preserved expression in non-neoplastic cells which acted as an internal positive control.

**Results:**

Loss of BAP1 expression was found in only 1 of 306 (0.33%) pancreatic ductal adenocarcinomas. This case was confirmed to demonstrate diffuse loss of expression throughout all neoplastic cells in multiple blocks, consistent with BAP1 loss being an early clonal event. All other cases demonstrated positive expression of BAP1.

**Conclusion:**

We conclude that, in contrast to intrahepatic cholangiocarcinoma, loss of expression of BAP1 occurs very rarely in pancreatic ductal adenocarcinoma. Therefore BAP1 inactivation is unlikely to be a frequent driver abnormality in pancreatic adenocarcinoma.

## Introduction

Pancreaticobiliary carcinomas may be classified as either intrahepatic or extrahepatic [1,2]. Approximately 90% of all pancreatic neoplasms originate from stem cells in the pancreatic ducts and are classified as pancreatic ductal adenocarcinoma (PDAC) [3]. Known risk factors for PDAC include smoking, diabetes, chronic pancreatitis, hepatic cirrhosis, diets high in fat and/or cholesterol, and a family history of the disease [2]. PDAC is one of the most prevalent and lethal cancers in the world with a 5-year survival rate of 5% [2,4–6]. This survival rate has shown the least improvement in the past 4 decades relative to other malignancies [6]. To date, surgical resection has been the sole potentially curative treatment. However, only approximately 20% of patients are considered operable and surgery can be associated with significant morbidity [4,5,7]. Patients undergoing surgery with or without adjuvant chemotherapy have an increased 5-year survival of between 20–25%, with a median survival of 1–2 years [7]. However in the majority of patients the cancer will recur and metastasize within 2 years of surgery [4]. The remaining 80% of patients who are inoperable have either locally advanced or metastatic disease at presentation and palliative chemotherapy is the only potential treatment [5,7]. In view of this, it would be beneficial to have a genomic biomarker which can be detected much earlier in this disease, perhaps to allow earlier detection or targeted intervention [2,4,5].

BRCA associated protein-1 (BAP1) is a 90kDa ubiquitous nuclear carboxy-terminal hydrolase, the gene for which is located on chromosome 3p21.1.[8] BAP1 has recently been reported to have a tumour suppressor role by way of transcription regulation, chromatin modification, and DNA damage response [9], and the *BAP1* gene has been shown to conform to Knudson’s classic two-hit model, whereby biallelic inactivation leads to tumorigenesis with or without germline mutation [8]. Biallelic inactivation and mutations of *BAP1* have been associated with a number of malignancies, including cutaneous melanocytic melanomas [10,11], mesothelioma [9,11–13] and renal cell carcinomas [9,14].

Germline *BAP1* mutation has now been demonstrated to be associated with an autosomal dominant hereditary tumour predisposition syndrome (Online Mendelian Inheritance in Man #614327). This syndrome is now accepted to include uveal and cutaneous melanoma, malignant mesothelioma, lung adenocarcinoma, meningioma, and renal cell carcinoma, and there are suggestions that other tumours including breast, ovarian, colon, and prostate cancers may be associated [4,8,9,11,12,14–21]. However, currently there is limited data on BAP1 expression in the pancreatobiliary system [16].

Given the finding that somatic *BAP1* mutations have been reported in a significant proportion of intrahepatic cholangiocarcinomas (CCA) [22,23,34], it is possible that cancers arising from *BAP1* mutations occur in the pancreas, considering the morphological similarity and anatomical proximity of the entire pancreatobiliary system. We therefore sought to assess the incidence and clinical significance of loss of BAP1 expression in PDAC through immunohistochemical staining.

## Methods

The electronic database of the department of Anatomical Pathology Royal North Shore Hospital was searched for cases of pancreaticobiliary carcinoma diagnosed between January 1992 and December 2014 with material available in archived formalin fixed paraffin embedded (FFPE) blocks. Adenocarcinomas of the ampulla of Vater and duodenum, extrahepatic cholangiocarcinomas, intraductal papillary mucinous neoplasms, neuroendocrine tumours, mucinous and serous cystadenomas, and solid pseudopapillary neoplasms were excluded. All cases were re-staged according to the 7^th^ edition of the American Joint Committee on Cancer staging manual [24].

**Fig 1:**
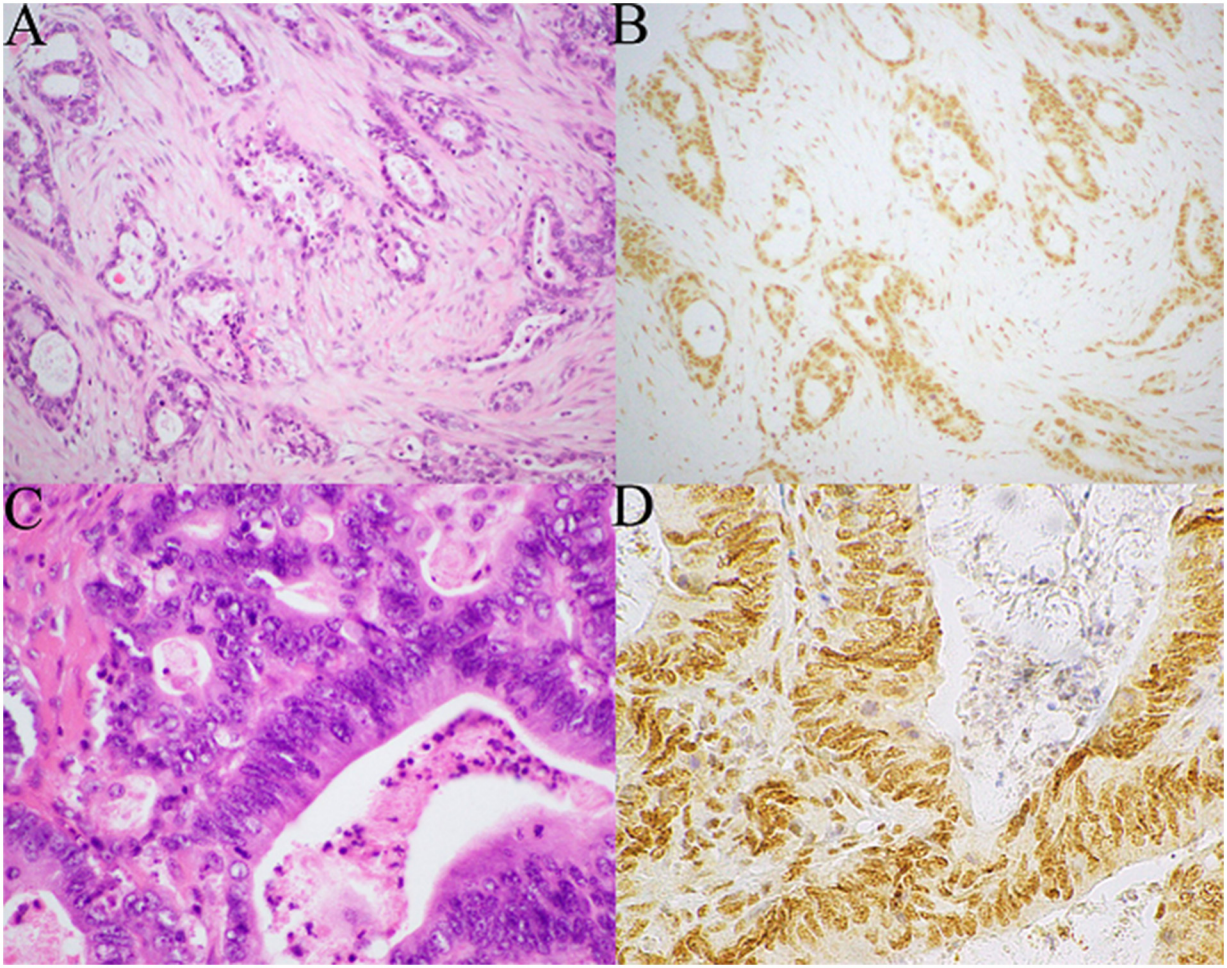
Serial H&E (A,C) and BAP1 IHC (B, D) stained sections of pancreatic ductal adenocarcinomas which demonstrate positive immunohistochemical staining for BAP1. All the neoplastic and non-neoplastic cell demonstrate diffuse strong nuclear staining (Original magnification A,B 100x, C,D 400x).

**Fig 2:**
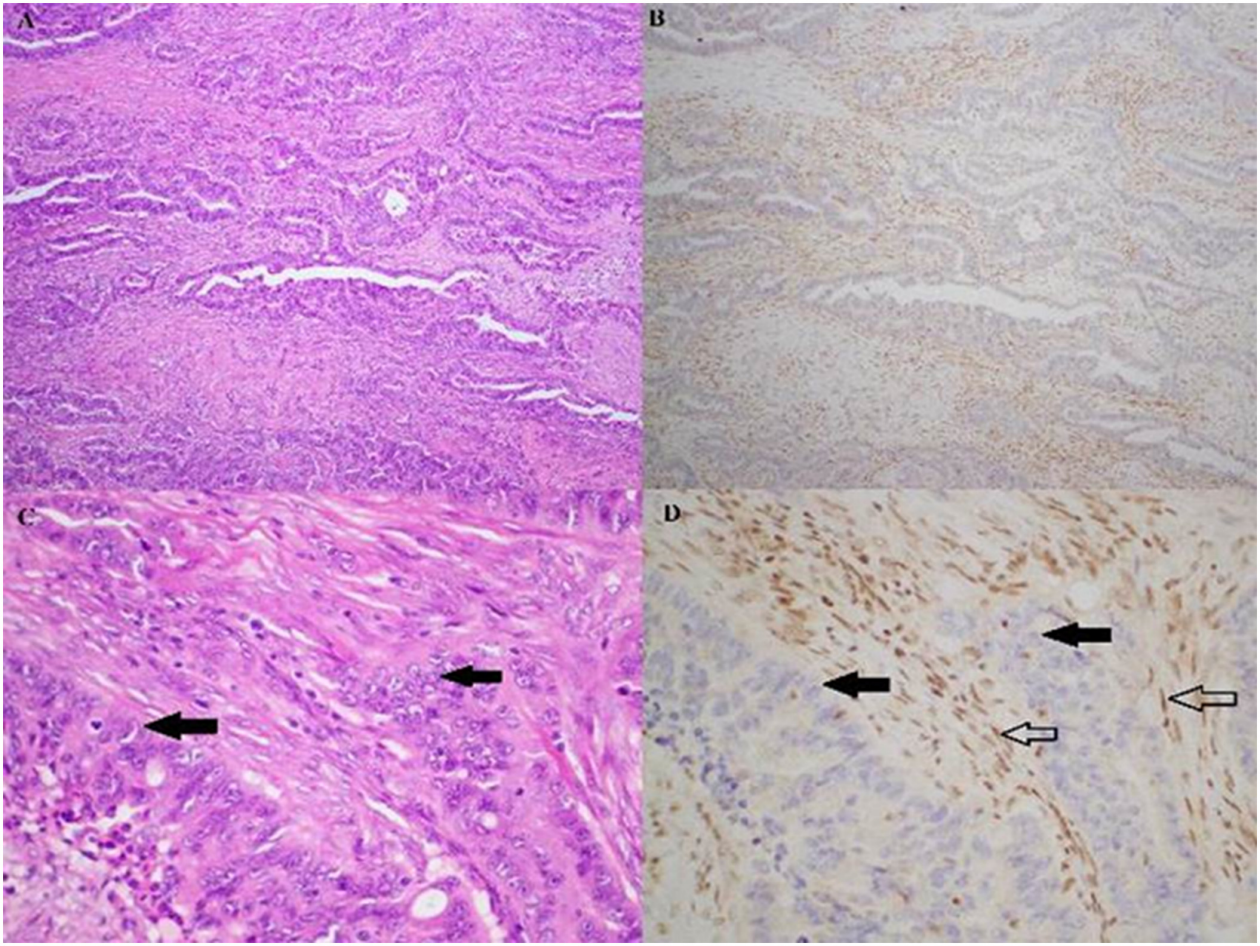
Haematoxylin & eosin (A, C) and BAP1 immunohistochemistry (B, D) stained sections from the sole pancreatic ductal adenocarcinoma patient who expressed loss of nuclear BAP1. In this case, the exocrine cells (solid arrows) clearly lacked the brown BAP1 staining, and the non-neoplastic endothelial cells serve as the internal positive controls (hollow arrows). Magnifications: A, B 100x; C, D 400x.

Tissue microarrays (TMAs) were constructed containing two 1mm diameter cores of tumour. Immunohistochemistry (IHC) for BAP1 was performed on this TMA using a mouse monoclonal antibody at a 1:200 dilution (clone C-4, cat no sc-28383, Santa Cruz Biotechnology, USA) after heat-induced antigen retrieval for 30 minutes at 97°C. Scoring of BAP1 staining was performed blinded by an experienced gastrointestinal pathologist (AG). Positive staining was defined as any nuclear staining in neoplastic cells (Fig 1). Negative staining was defined as the absence of nuclear staining in all neoplastic cells in the presence of positive staining in non-neoplastic cells such as lymphocytes and endothelial cells which acted as internal positive controls (Fig 2). If the neoplastic cells were negative, but there was no good internal positive control in non-neoplastic cells, the staining was considered equivocal and repeated on whole sections. All negative and equivocally staining cases were repeated on whole sections.

Overall survival data was obtained from hospital medical records and from publicly available death notices. Survival was determined by Kaplan-Meier analysis. Overall survival was defined as the duration from the original tissue diagnosis through to death or 31 May 2015. The impact of age, gender, tumour stage and grade, vascular invasion, perineural invasion, and lymph node metastasis on overall survival outcome was assessed using multivariate Cox regression. Univariate Cox regression compared the impact of each individual variable on overall survival. Statistical significance was assumed at p < 0.05. All statistical analyses were carried out using IBM Statistical Packages for the Social Sciences (SPSS) Statistics v22. The study was approved by the Northern Sydney Local Health District Medical Ethics Review Board (ref: LNR/13/HAWKE/424). Unidentified data from which the statistical analyses were performed has been supplied in S1 Table.

## Results

Between January 1992 and December 2014, 700 patients were diagnosed with PC and extrahepatic CCA and underwent surgical resection with curative intent. After the exclusion criteria were applied, tissue blocks of 306 patients diagnosed with definitive PDAC were available for the creation of the TMA and IHC for BAP1. The patients’ clinical and pathological details are summarized in Table 1. Briefly, the mean age at diagnosis was 68 years (range: 28–87 years), and 138 (45.3%) patients were male. Staging showed 89% of patients were stage II at the time of resection, with stages I, III and IV each contributing 2.6%, 3.0% and 5.6%, respectively. Vascular invasion was present in 155 (60.3%) tumours. Perineural invasion occurred in 204 (74.5%), and lymph node metastasis was found in 191 (62.8%) tumours.

**Table 1:** Clinical and pathological characteristics of 306 pancreatic adenocarcinoma patients.

Variable	N (%)
Age at diagnosis in years, mean (range)	67.8 (28–87)
Survival in months, median (range)	26.4 (21.7–31.1)
Gender	
Male	138 (45.1)
Female	168 (54.9)
Overall stage	
Stage I	8 (2.6)
Stage II	271 (88.6)
Stage III	9 (2.9)
Stage IV	17 (5.6)
Tumour Grade	
G1	17 (5.6)
G2	199 (65.0)
G3	79 (25.8)
G4	7 (2.3)
Vascular Invasion	
No vascular invasion	102 (33.3)
Vascular invasion present	155 (50.7)
Presence of perineural growth	
No perineural growth	70 (22.9)
Perineural growth present	204 (66.7)
Lymph node metastasis	
No lymph node metastasis	113 (36.9)
Lymph node metastasis present	191 (62.4)
Tumour size in mm, mean (range)	36.7 (3–100)

**Fig 3:**
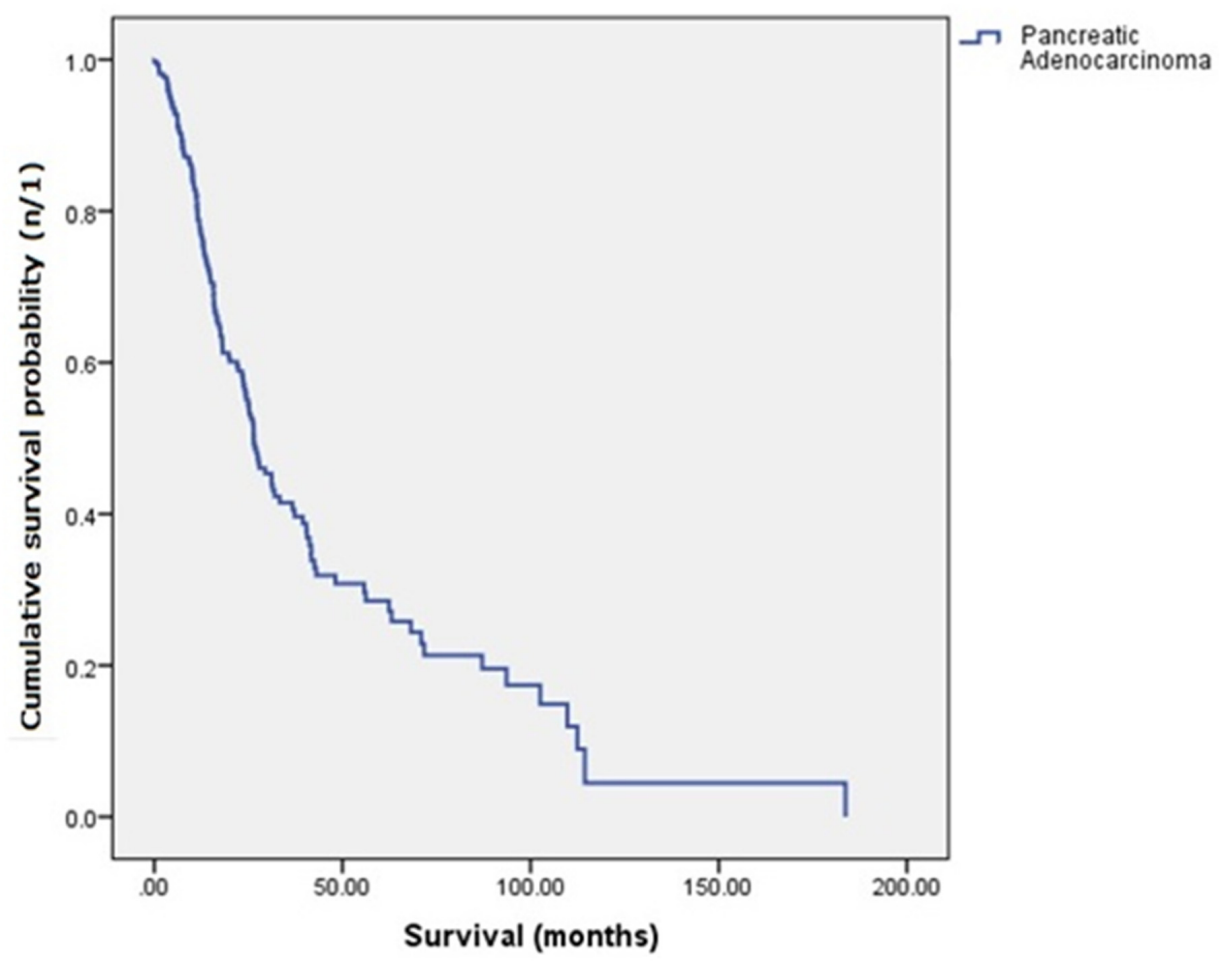
Kaplan-Meier survival curve for 306 pancreatic ductal adenocarcinoma patients.

The median overall survival for the cohort was 26.4 months (95% Confidence Interval [CI], range: 21.7–31.1 months) with a 28.0% 5-year survival rate. The Kaplan-Meier survival curve is presented in Fig 3.

Table 2 details the univariate and multivariate Cox regression proportional hazards analysis of the cohort showing hazard ratios (HR) for death. The analysis is based on the 209 of the 306 patients for whom complete details were available for all variables. There was no significant difference in survival in terms of age at diagnosis. Female patients trended towards better survival than males, however this did not reach statistical significance on univariate (HR 0.77; 95% CI 0.55–1.08; p = 0.13) or multivariate (HR 0.76; 95% CI 0.50–1.15; p = 0.20) analysis. Higher grade predicted worse survival outcomes, although this only reached statistical significance for Grade 3 tumours, which were associated with increased mortality in both univariate (HR 2.85; 95% CI 1.37–5.96; p < 0.01) and multivariate (HR 3.16; 95% CI 1.12–8.89; p = 0.0.) analysis. The presence of vascular invasion (HR 1.85; 95% CI 1.12–3.05; p = 0.02), lymph node metastasis (HR 1.86; 95% CI 1.14–3.03; p = 0.01), and increasing tumour size (HR 1.02; 95% CI 1.02–1.04; p < 0.01) all indicated significantly worse survival on multivariate analysis, while the presence of perineural growth was not a significant predictor of survival outcome (p = 0.995).

**Table 2:** Univariate and multivariate Cox regression proportional hazards analysis of 306 pancreatic adenocarcinoma patients.

Variable	Univariate cox regression; HR (95%CI), p-value	Multivariate cox regression; HR (95%CI), p-value
Age at diagnosis	1.02 (0.995–1.04), 0.14	1.02 (0.995–1.04), 0.12
Gender		
Male	1.00	1.00
Female	0.77 (0.55–1.08), 0.13	0.76 (0.50–1.15), 0.20
Overall stage		
Stage I	1.00	1.00
Stage II	2.24 (0.82–6.14), 0.12	0.79 (0.17–3.71), 0.76
Stage III	1.60 (0.40–6.45), 0.51	0.24 (0.03–1.82), 0.17
Stage IV	2.36 (0.68–8.19)	0.65 (0.11–3.94), 0.61
Tumour Grade		
G1	1.00	1.00
G2	1.89 (0.94–3.80), 0.72	2.13 (0.80–5.64), 0.13
G3	**2.85 (1.37–5.96), <0.01**	**3.16 (1.12–8.89), 0.03**
G4	1.46 (0.39–5.44), 0.57	1.34 (0.26–6.85), 0.72
Vascular Invasion	**2.08 (1.38–3.15), <0.01**	**1.85 (1.12–3.05), 0.02**
Presence of perineural growth	1.38 (0.90–2.12), 0.15	0.998 (0.59–1.70), 0.995
Lymph node metastasis	**1.53 (1.07–2.19), 0.02**	**1.86 (1.14–3.03), 0.01**
Tumour size	1.01 (1.00–1.02), 0.05	**1.02 (1.01–1.04), <0.01**

Only one case demonstrated negative immunohistochemical staining for BAP1. In this case all neoplastic cells in both the TMA and several whole sections of tumour demonstrated negative staining for BAP1 in the presence of an internal positive control (Fig 2). This case arose in a 64-year-old male who underwent surgical resection for a Stage I, Grade 3 tumour which did not exhibit any vascular or perineural invasion. At last follow-up, the patient was alive and disease free at 17.7 months after the initial surgery.

## Discussion

*BAP1* is a tumour suppressor gene which has been implicated in an autosomal dominant hereditary tumour predisposition syndrome associated with heterozygous germline mutation [8,9,16]. Previous studies have demonstrated the robustness and reliability of BAP1 IHC in detecting double hit inactivation/mutation in multiple tumours including uveal melanoma [25,26], cutaneous melanoma [10,21,27], malignant mesothelioma [16,28], and renal cell carcinoma [14]. This was the first known study which specifically investigated BAP1 IHC in a large cohort of PDAC patients. In this cohort, the median overall survival was 26.4 months (95% CI, range: 21.7–31.1 months) and the 5-year survival rate 28.0%. For patients suitable for surgical resection, this concurs quite closely with other studies which have estimated the median overall survival to range between 12 and 22 months and a 7–25% chance of survival beyond 5 years [4,5,7,29]

Loss of BAP1 IHC expression was observed in only 1 of the 306 (0.33%) PDAC cases tested. The Australian Pancreatic Cancer Genome Initiative, the International Cancer Genome Consortium, and several other studies, who have performed whole exome and genome sequencing on a number of PDAC specimens have yet to locate any BAP1 mutations or deletions in PDAC [4,30–32], which closely conforms with the results of this research. Furthermore, the only known study specifically targeting BAP1 mutations in PDAC likewise did not find any somatic BAP1 mutations [33].

This result is perhaps surprising given that BAP1 inactivation or loss of BAP1 expression has been detected using IHC and whole-exome sequencing in approximately 25% of the intrahepatic CCAs [22,23,34]. Besides their close anatomical proximity to one another, numerous reports suggest that the ventral pancreas shares many similarities with the extrahepatic bile duct, including aspects of embryogenesis [35,36]. Extrahepatic CCA in turn differs significantly from intrahepatic CCA in terms of biology, morphology, genetic mutations, expression profiling, and embryology, amongst other factors [35,36]. Our finding that BAP1 loss is rare in PDAC whilst it is common in intrahepatic cholangiocarcinoma further reinforces the concept that they are different diseases. In fact, in a pancreaticobiliary malignancy when the differential diagnosis may be between PDAC metastatic to the liver and intrahepatic CCA, loss of BAP1 IHC expression can be used to support a diagnosis of intrahepatic CCA.

For this study we chose to assess BAP1 immunohistochemistry with a binary approach (that is, as either positive or negative), rather than using a semiquantitative approach (looking at intensity of expression). This binary approach to BAP1 immunohistochemistry assessment is the dominant approach used by surgical pathologists in routine clinical practice and has shown both excellent interobserver concordance and to correlate very well with molecular testing in a variety of tumour types [9,10,12,26,28,34,37–44]. We emphasize that using this approach a tumour is only considered BAP1 negative by IHC if all neoplastic cells show loss of expression of BAP1 and there is a positive internal control in non-neoplastic cells.

In conclusion, we present the first study of BAP1 IHC in a large cohort of PDAC patients. The incidence of loss of BAP1 IHC expression was just 0.33%, which confirms recent next-generation sequencing analyses which did not find BAP1 mutations or inactivation in large PDAC cohorts. That is, BAP1 loss occurs extremely rarely in PDAC.

## Supporting Information

S1 Table Data points for patient characteristics of 306 patients with pancreatic adenocarcinoma. (XLSX)
